# Multi-parameter optimization of recombinant protein secretion in *Bacillus subtilis:* a review

**DOI:** 10.1186/s12934-026-03155-y

**Published:** 2026-10-17

**Authors:** Askar Kholikov, Khushnut Vokhidov, Barno Alimova, Ozodakhon Pulatova, Yulong Zhang, Shihui Yang, Guimin Zhang, Yihua Chen, Akhmadzhan Makhsumkhanov

**Affiliations:** 1 Institute of Microbiology, Academy of Sciences of the Republic of Uzbekistan, Tashkent, 100128 Uzbekistan; 2 Institute of Biochemistry, Heinrich-Heine University Düsseldorf, 40225 Düsseldorf, Germany; 3 Institute of Microbiology, Chinese Academy of Sciences, Beijing, 100101 China; 4 University of Chinese Academy of Sciences, Beijing, 100049 China; 5 State Key Laboratory of Biocatalysis and Enzyme Engineering, School of Life Sciences, Hubei University, Wuhan, 430062 China; 6 State Key Laboratory of Green Biomanufacturing, College of Life Science and Technology, Beijing University of Chemical Technology, Beijing, 100029 China

**Keywords:** *Bacillus subtilis*, Recombinant protein secretion, Signal peptide and promoter engineering, Ribosome binding site, Sec and Tat pathway, Industrial enzyme

## Abstract

*Bacillus subtilis* is widely recognized as one of the most robust and versatile hosts for recombinant protein production. This review provides a comprehensive overview of the protein secretion pathway in *B. subtilis*, detailing its mechanistic basis and key limiting steps. We discuss the structural features of signal peptides (SPs) that govern efficient translocation and highlight recent advances in de novo promoter and signal peptide engineering. A particular focus is placed on the integration of artificial intelligence (AI) to predict and design optimal genetic elements. Drawing on findings from combinatorial and multi-parameter studies, we propose that future engineering strategies should move beyond maximizing individual parameters. Instead, achieving a dynamic balance across transcriptional, translational and secretion layers is essential to sustain high productivity while minimizing cellular secretion stress. This review offers a roadmap for systems-level engineering of *B. subtilis* as a next-generation protein production platform.

## Introduction

 Recombinant protein production in bacterial hosts remains a cornerstone of modern biotechnology, enabling the large-scale manufacture of enzymes, biopharmaceuticals and industrial biocatalysts that collectively represent a multi-million dollar global market [1, 2]. Among microbial platforms, bacteria offer clear advantages in terms of rapid growth, genetic tractability and cost-effective cultivation. Traditionally, *E. coli* has dominated as the primary expression host, however, its limitations particularly with respect to protein secretion [3], endotoxin contamination [4], and improper folding [5] of complex proteins have driven increasing interest in alternative bacterial systems. In this context, *B. subtilis* has emerged as one of the most attractive Gram positive hosts for recombinant protein secretion [6, 7].

*B. subtilis* expression systems are widely used for the production of recombinant proteins, with estimates of their contribution to the global enzyme market varying from 17% of total commercial enzyme production in 2014 [8] to approximately 50% based on incomplete market statistics [9]. Advances in genome editing technologies have enabled fine control over transcriptional and translational processes, allowing tailored expression of heterologous genes [10, 11]. Nevertheless, a major bottleneck in bacterial production remains downstream processing, which can account for up to 80% of total production costs [12]. Intracellular expression often necessitates cell disruption, complex purification procedures and refolding steps, especially when proteins accumulate as insoluble inclusion bodies. Gram positive bacteria such as *B. subtilis* secretion of recombinant proteins directly into the culture medium is highly desirable, as it simplifies purification, improves product quality and enhances overall process economics [2, 13].

*B. subtilis* offers several key advantages over *E. coli* as a recombinant protein production host. One of the most important is its exceptional capacity for protein secretion. *B. subtilis* natively secretes enzymes such as, proteases [14], lipases [15], amylases and cellulases [16] at high levels, making it a natural choice for extracellular enzyme production (Table 1). Proteins exported via the Sec or Tat pathways are released directly into the culture medium, facilitating efficient recovery [17]. In addition, *B. subtilis* is classified as “Generally Recognized as Safe” (GRAS), which is a critical advantage for applications in the food, feed and pharmaceutical industries [18]. From a bioprocessing perspective, *B. subtilis* is robust, grows to high cell densities and tolerates diverse environmental conditions. These properties, combined with its secretion capacity, have positioned *B. subtilis* as a preferred host for industrial enzyme production and an increasingly important alternative to *E. coli* for recombinant protein expression [6, 19, 20].

**Table 1:** Overview of industrially important enzymes produced by *B. subtilis*, including their applications, secretion pathway and best performing typical signal peptides (SPs)

№	Enzyme	Industrial Application	Secretion Pathway	Typical SP	Reported Yield^a^	References
1	α-Amylase	Starch, detergents and pharmaceutical industry	Sec	AprE	1834 (U/ml)	[21]
2	Protease	Food, feed, detergents and leather industry	Sec	Bschi	8253 (U/ml)	[22]
3	Lipase	Food industry, bio-fuels	Sec	LipA	0.57 (U/ml)	[23]
4	Xylanase	Animal feed, pulp and paper	Sec	SamyQ	4.28 (U/ml)	[24]
5	Phytase	Animal feed	Sec	pbp	⁓4200 (µmol/L)	[25]
6	Pullulanase	Starch processing, animal feed, bio-fuels	Sec	chiA	⁓380 (U/ml)	[26]
7	Isoamylase	Food industry	Sec	NprB	234 (mg/L)	[27]
8	Nattokinase	Food industry Pharmaceutical	Other	YqxM	235.2 (FU/mL)	[28]
9	Keratinase	Feather, wool processing, animal feed	Sec	LipA	830.91 (U/ml)	[29]
10	L-asparaginase	Food and pharmaceutical industry	Sec	wapA	28.91 (U/mL)	[30]
11	Laccase	Biofuels, paper and textile industries	Sec	wapA	373.1 (U/mL)	[31]
12	Amidase	Biotransformation	Sec	Pac	135.58 (U/mL)	[32]
13	β-Galactosidase	Food industry	Tat	PhoD	1.15 (U/mL)	[33]

^a^ Yields are reported using the original study units and are not directly comparable across enzymes. FU/mL, fibrinolytic units per milliliter, denotes nattokinase activity

Secreted enzymes produced by *B. subtilis* play a central role in multiple industrial sectors, including food and beverage processing, detergents, textiles, paper, biofuels and pharmaceuticals. For example, α-amylases are widely used in starch processing, baking, brewing and bioethanol production [34–36], proteases are key components of detergent formulations and are extensively applied in feed processing, waste management and protein hydrolysis [37–39]. Xylanases are the key enzymes for animal food manufacturing, pulp bleaching, detergent, biofuels, wine and baking industry [40–42]. L-asparaginase is a clinically important enzyme used as a chemotherapeutic agent for the treatment of acute lymphoblastic leukemia by depleting L-asparagine, an amino acid required for leukemic cell survival. It is also applied in the food industry to reduce acrylamide formation during the production of heat-processed foods [43, 44]. Laccases are important oxidases with widespread applications in sustainable bioenergy and biomaterial production [45]. The demand for enzymes with improved stability, activity and specificity has driven intensive efforts in protein engineering and host optimization. Efficient secretion is often a decisive factor determining the commercial viability of an enzyme, as it directly impacts yield, purity and production cost. Consequently, optimizing recombinant protein secretion in *B. subtilis* has become a major research focus, encompassing SP engineering, translational control via ribosome binding sites (RBS) and host strain engineering [9, 10].

This review focuses on current strategies for optimizing recombinant protein secretion in *B. subtilis*, including SP engineering, translational control via promoter and RBS optimization, and systems-level multi-parameter approaches. It offers practical guidance for developing efficient secretion platforms for industrial and biopharmaceutical applications.

### Protein secretion pathways in *B. subtilis*

**Fig. 1:**
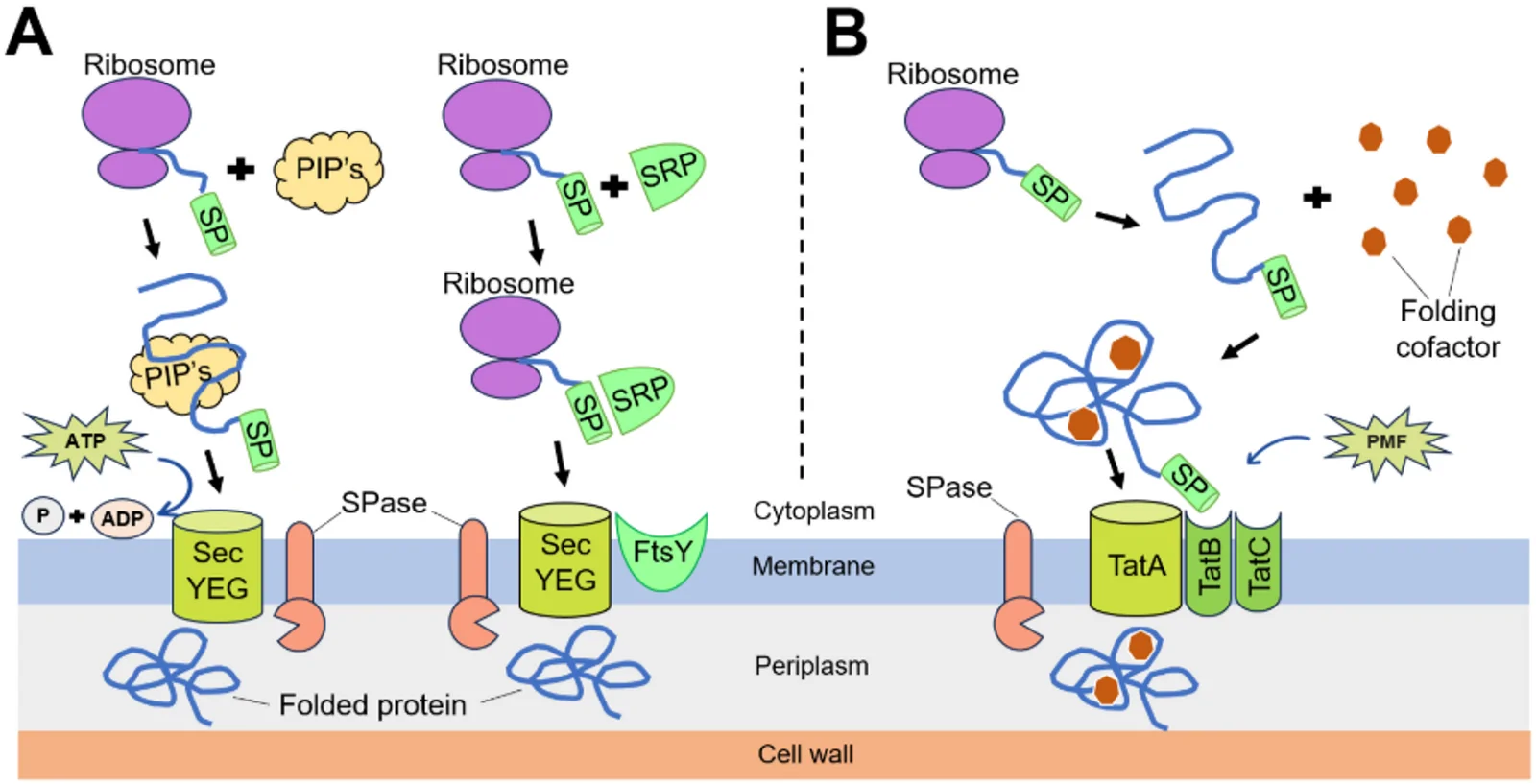
Schematic representation of protein secretion pathways in *B. subtilis*. (**A**) Sec-dependent protein export pathway. In the post-translational pathway, newly synthesized preproteins containing N-terminal signal peptides (SPs) are maintained in an unfolded state by post-translational interacting proteins (PIP’s), which drives their translocation through the SecYEG protein-conducting channel. In the co-translational pathway, SPs emerging from the ribosome are recognized by the signal recognition particle (SRP), which targets the ribosome-nascent chain complex to the membrane through interaction with the FtsY receptor, followed by transfer of the substrate to the SecYEG channel. ATP hydrolysis provides the energy required for translocation. (**B**) Tat-dependent protein export pathway. Folded preproteins containing signal peptides with a twin-arginine (RR) motif are recognized by the TatBC receptor complex. Recruitment and assembly of TatA oligomers facilitate substrate translocation across the cytoplasmic membrane using the proton motive force (PMF) as the energy source. In both pathways, signal peptides are cleaved by signal peptidase (SPase) at the membrane, releasing mature proteins into the extracellular environment. Following cleavage, the signal peptide is released from the mature protein and subsequently degraded within the membrane

Protein secretion in *B. subtilis* is a highly coordinated and tightly regulated process that enables the transport of proteins from the cytoplasm to the extracellular environment. In *B. subtilis*, protein export is primarily mediated by two major pathways: the general secretory (Sec) pathway and the twin-arginine translocation (Tat) pathway [2, 46, 47]. Both systems rely on N-terminal SP that target preproteins to the cytoplasmic membrane [48], but they differ fundamentally in their substrate requirements, energy usage and secretion mechanisms [17] (Fig. 1). Understanding the principles, strengths and limitations of these pathways is essential for the rational design of efficient recombinant protein secretion strategies.

### Sec pathway

The Sec pathway is the dominant and most extensively studied protein secretion route in *B. subtilis* and is responsible for the export of the vast majority of secreted and cell wall-associated proteins. Proteins translocated via the Sec system are synthesized as precursor proteins (preproteins) containing an N-terminal SP composed of three conserved regions: a positively charged N-region, a hydrophobic H-region and a SP cleavage site in the C-region [49]. In the Sec-dependent pathway, proteins are typically exported in an unfolded or partially folded state. During or shortly after translation, preproteins are targeted to the Sec translocon (SecYEG) either co-translationally via the signal recognition particle (SRP) that is targeted to FtsY receptor [50], or post-translationally interacting proteins (PiP’s) [2] such as SecA [51]. SecA plays a central role as an ATP-driven motor protein that pushes the preprotein through the SecYEG channel in the cytoplasmic membrane. Once translocation is complete, the SP is cleaved by SPase, releasing the mature protein into the extracellular space. For recombinant protein production, the Sec pathway is generally preferred due to its high capacity and broad substrate range [52]. However, overloading the Sec machinery through excessive expression or poorly optimized SP can lead to secretion stress, accumulation of precursor proteins and increased degradation by quality-control proteases. Consequently, fine-tuning of expression levels and SP properties is critical for maximizing secretion efficiency through this pathway [53].

### Tat pathway

The Tat pathway represents an alternative secretion route in *B. subtilis*, specialized for the export of fully folded proteins and protein complexes [46, 54]. Tat SPs contain a highly conserved twin-arginine motif (RR) in their N-region, which distinguishes them from Sec-targeted substrates. Unlike the Sec system, the Tat pathway transports proteins in a folded state, which is particularly advantageous for proteins requiring the incorporation of cofactors, metal ions, or complex prosthetic groups in the cytoplasm prior to secretion. The Tat machinery in *B. subtilis* composed of two parallel systems, TatA and TatC, which form a transient translocation pore driven by the proton motive force rather than ATP hydrolysis [55–57]. Despite these advantages, the Tat pathway has several limitations that restrict its widespread use for recombinant protein secretion. In *B. subtilis*, only a limited number of native proteins (PhoD, QcrA, YkuE and YwbN) are known to be exported through the Tat pathway, reflecting its substantially narrower substrate spectrum compared with the Sec pathway [58]. Furthermore, secretion efficiency strongly depends on the compatibility between the Tat signal peptide and the cargo protein. For example, the TorA signal peptide successfully mediates GFP export in *Corynebacterium glutamicum* but fails to produce active extracellular GFP in *B. subtilis* [59]. Likewise, the YwbN signal peptide has been shown to redirect subtilisin, a typical Sec-dependent secretory protein, through the Tat pathway, whereas another Sec substrate, α-amylase, remained Tat-independent despite carrying the same signal peptide [59]. These findings demonstrate that Tat-mediated secretion is highly dependent on the compatibility between the signal peptide and the cargo protein, making secretion outcomes difficult to predict and optimize. Together with its relatively low transport flux compared with the Sec pathway, these characteristics currently limit the broader industrial application of Tat-dependent secretion. As a result, while the Tat pathway can be valuable for specific proteins that cannot be secreted in an unfolded state, it is generally considered a secondary option for recombinant protein production in *B. subtilis*. Most industrial and research applications therefore focus on optimizing Sec-dependent secretion through SP engineering, translational control and host strain modification, which are discussed in subsequent sections of this review.

### Structure and function of signal peptides

**Fig. 2:**
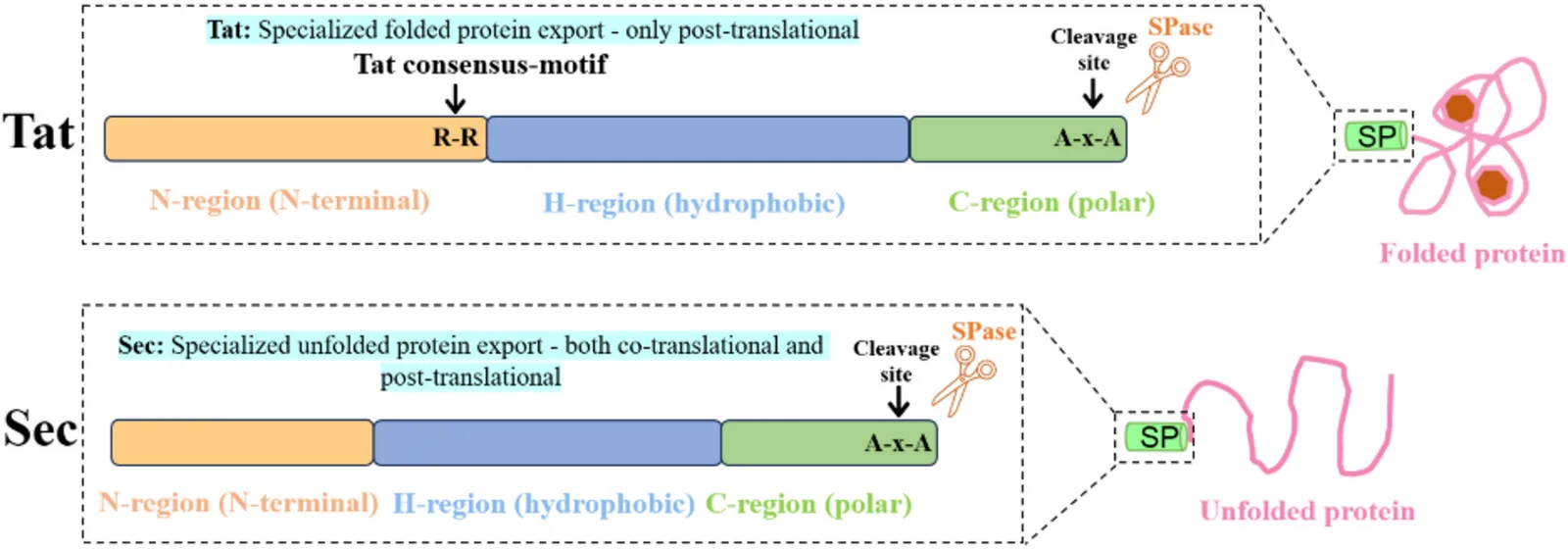
Structural organization and functional features of Sec- and Tat-dependent SPs. Both Sec and Tat SPs share a conserved tripartite architecture consisting of an N-region, H-region and C-region. The N-region is enriched in positively charged residues that contribute to membrane targeting, whereas the hydrophobic H-region facilitates interaction with the membrane and the translocation machinery. The C-region contains the signal peptidase (SPase) recognition site, typically characterized by a conserved A-x-A motif, where cleavage occurs to release the mature protein. Tat SPs are distinguished by the presence of a conserved twin-arginine (RR) motif located at the N-/H-region boundary, which enables recognition by the Tat translocase and transport of fully folded proteins. In contrast, Sec SPs generally lack the RR motif and direct unfolded proteins through the Sec pathway

SPs are short N-terminal amino acid sequences that direct newly synthesized proteins to the cellular secretion machinery. In *B. subtilis*, SPs are essential determinants of whether a recombinant protein enters the Sec or Tat export pathways and strongly influence secretion efficiency, processing, and final yield [60, 61]. Although SPs are typically removed during secretion and are not part of the mature protein, their sequence composition and structural features play a critical role in targeting, membrane translocation and SPase recognition [49]. Despite considerable sequence diversity, most SPs share a conserved tripartite architecture consisting of an N-region, an H-region and a C-region [62] (Fig. 2). Subtle variations in the length, charge and hydrophobicity of these regions can profoundly affect secretion performance, making SP engineering one of the most powerful tools for optimizing recombinant protein secretion in *B. subtilis.*

The N-region is located at the extreme N-terminus of the SP and it is enriched in positively charged residues, mainly lysine (Lys) and arginine (Arg), which are thought to promote correct orientation of the SP in the cytoplasmic membrane according to the “positive-inside rule”. These positive charges facilitate interaction with the negatively charged phospholipid head groups of the membrane and contribute to efficient targeting of the preprotein to the Sec or Tat translocon [62]. In Tat SPs, the N-region also contains the hallmark RR motif, which is essential for recognition by the Tat machinery [63]. The H-region follows the N-region and is the most hydrophobic segment of the SP, typically consisting of 7–15 predominantly hydrophobic amino acids such as leucine, isoleucine, valine and alanine [62]. The hydrophobicity and length of the H-region are critical for insertion of the SP into the cytoplasmic membrane and for stable interaction with the Sec translocon. In *B. subtilis*, SPs with moderately strong hydrophobic H-region often perform better than extremely hydrophobic ones, as excessive hydrophobicity can lead to membrane trapping or inefficient processing [62, 64]. Fine-tuning the H-region is therefore a common strategy to balance membrane insertion with productive translocation.

The C-region is located immediately upstream of the SP cleavage site and this region is more polar and less hydrophobic than the H-region and contains the recognition motif for SPase. Following translocation across the cytoplasmic membrane, SPs are removed by SPase, which are essential enzymes in *B. subtilis*. Most secreted proteins are processed by type I SPase, which recognize the C-region cleavage motif and cleave the SP from the mature protein [2, 65]. This cleavage step is crucial, as inefficient or incomplete processing can result in membrane-anchored intermediates, reduced extracellular accumulation or increased susceptibility to proteolytic degradation [66].

For recombinant protein production in *B. subtilis*, careful selection or rational design of SPs with optimized N-, H- and C-regions is essential [67]. In general, SPs with balanced N-region charge, moderate H-region hydrophobicity and an intact A-x-A cleavage motif tend to support efficient secretion, although optimal performance remains highly cargo-dependent. Empirical screening of native *Bacillus* SPs, combined with sequence engineering and computational prediction tools, has proven highly effective for enhancing the secretion of industrially relevant enzymes such as amylase, xylanase and cutinase [68]. Understanding the structural and functional principles of SPs therefore provides a critical foundation for the advanced secretion optimization strategies discussed in the following sections.

### Signal peptide engineering for enhanced secretion

Efficient secretion of recombinant proteins in *B. subtilis* critically depends on the choice and optimization of the SP. Even when the same target protein, promoter and host strain are used, different SPs can result in secretion levels that vary by orders of magnitude. This strong context dependency reflects the complex interplay between SP sequence, translational kinetics, membrane targeting, translocation efficiency and post-translocational processing. Consequently, SP engineering has become a central strategy for improving extracellular production of industrially relevant enzymes in *B. subtilis*.

### Natural SPs used in B. subtilis

A widely adopted approach to enhance secretion efficiency is the use of SPs derived from native *B. subtilis* or closely related *Bacillus* species that naturally secrete large amounts of proteins. These natural SPs have evolved to function efficiently with the Sec machinery and are often highly effective when fused to heterologous proteins. Among the most extensively used SPs is that of AmyQ, the α-amylase from *B. amyloliquefaciens* [64, 69, 70]. The AmyQ SP is considered a benchmark for secretory expression in *B. subtilis* and has been successfully applied to the secretion of numerous recombinant enzymes [71]. Another commonly used SP originates from AprE, the major alkaline serine protease (subtilisin) of *B. subtilis*. The AprE SP is particularly effective for the secretion of proteases and other enzymes with similar folding and secretion kinetics. Due to the natural high-level expression of AprE, its SP is often combined with strong promoters for industrial enzyme production [72]. SPs derived from YoaW [73] and LipA (lipase) [15, 74] have also been widely explored. These SPs exhibit diverse sequence features and secretion behaviors, making them valuable components in SP libraries. Notably, the YoaW SP has gained attention for its ability to enhance secretion of certain heterologous proteins that perform poorly with more commonly used SPs, underscoring the importance of empirical testing. Overall, no single “universal” SP exists that performs optimally for all proteins. The secretion efficiency is highly protein-specific and native SPs provide a rich and functionally validated starting point for secretion optimization in *B. subtilis*.

### SP library screening

Given the strong context dependency of SP performance, systematic screening strategies have been developed to identify optimal SPs for a given target protein. Rather than relying on rational design alone, many studies employ combinatorial and high-throughput approaches to rapidly explore a wide range of SP variants.

Experimental SP libraries are typically constructed by fusing a set of natural or synthetic SPs upstream of the same target gene, while keeping all other genetic elements constant. Libraries may consist of dozens to hundreds of SPs derived from *B. subtilis* [8, 75, 76] and related *Bacillus* species [52, 59], or may include engineered variants with systematic modifications in the N-, H- or C-regions [77]. Advances in DNA synthesis and cloning technologies have greatly simplified the generation of such libraries. Modular cloning systems and standardized secretion vectors allow rapid assembly and parallel testing of SP-protein fusions. In some cases, semi-rational libraries are designed by varying specific physicochemical parameters, such as H-region hydrophobicity, N-region charge and N-terminal sequence composition, to probe their influence on secretion efficiency [60, 74]. The secretion performance of an SP is determined not only by individual features but also by the synergistic interplay among regional length, charge distribution, and hydrophobicity. For example, an appropriate N-region charge-to-length ratio, optimized H-region hydrophobicity, and favorable C-region processing characteristics can improve signal peptide recognition and translocation efficiency [60]. Furthermore, subtle modifications within the N-terminal region, including the first few amino acids of the SP, can significantly influence protein expression and secretion capacity of heterologous proteins [74]. Therefore, rational SP engineering requires optimization of multiple structural parameters rather than modification of a single region.

These libraries enable the identification of SPs that are optimally matched to the folding kinetics and secretion requirements of the target protein. The success of SP screening depends on reliable and scalable assays for detecting secreted proteins. High-throughput secretion assays are commonly based on enzymatic activity measurements, immunodetection or reporter systems [75, 77]. For enzymes such as amylases and proteases, plate-based assays using chromogenic or fluorogenic substrates provide a rapid and cost-effective means to screen large numbers of clones. Halo formation on starch or casein agar plates, for example, is frequently used as a qualitative or semi-quantitative indicator of secretion efficiency. More quantitative high-throughput approaches include microtiter plate assays measuring extracellular enzyme activity or protein concentration, as well as fluorescence-based reporters that are only active or detectable upon secretion [78].

By combining experimental SP libraries with high-throughput secretion assays, researchers can rapidly identify top-performing SPs that significantly enhance recombinant protein secretion in *B. subtilis*. For example, Vološen and colleagues demonstrated in *B. subtilis* that SP protein compatibility is highly specific, with only a limited number of SPs enabling efficient secretion (8/74 for P-pheromone and 3/74 for α-pheromone), emphasizing the need for systematic screening approaches [8]. Expanding on this, Zhang and co-workers performed a large-scale analysis of 244 native SPs and identified Sec pathway-associated peptides as key drivers of secretion efficiency, achieving substantially higher yields (up to ⁓75 mg/L) for a therapeutic fusion protein [79]. Collectively, these studies underscore that rational selection and high-throughput screening of SP libraries are essential strategies for optimizing Sec-dependent protein secretion and improving industrial bioproduction efficiency.

### In silico and AI-assisted SP design

While experimental screening remains the gold standard for identifying high-performance SPs [13], it is often labor-intensive and time-consuming. To complement wet-lab approaches, in silico prediction and more recently, AI-assisted design tools have become increasingly important for guiding SP selection and engineering in biotechnology [80, 81]. These computational strategies aim to predict secretion efficiency based on sequence features and to reduce the experimental search space by prioritizing promising candidates [82]. Several bioinformatic tools have been developed to identify SPs and predict their secretion pathways. Classical tools such as SignalP [81], DeepSig [83], Phobius [84] and PrediSi [85] are widely used to detect SPs and discriminate between Sec- and Tat-dependent secretion based on conserved sequence motifs and physicochemical properties. Although these tools were originally designed for SP identification rather than performance ranking, they are invaluable for validating SP architecture and cleavage sites prior to experimental testing. More advanced platforms integrate machine learning algorithms trained on large datasets of experimentally validated SPs. Recent versions of SignalP, for example, employ deep neural networks that improve cleavage site prediction and pathway classification accuracy [81] (Table 2). AI-assisted approaches, including supervised learning and neural network models, are increasingly being applied to capture these complex, non-linear relationships. By training models on experimental datasets, it is possible to predict secretion performance for novel SPs and even generate entirely new sequences with optimized properties.

**Fig. 3:**
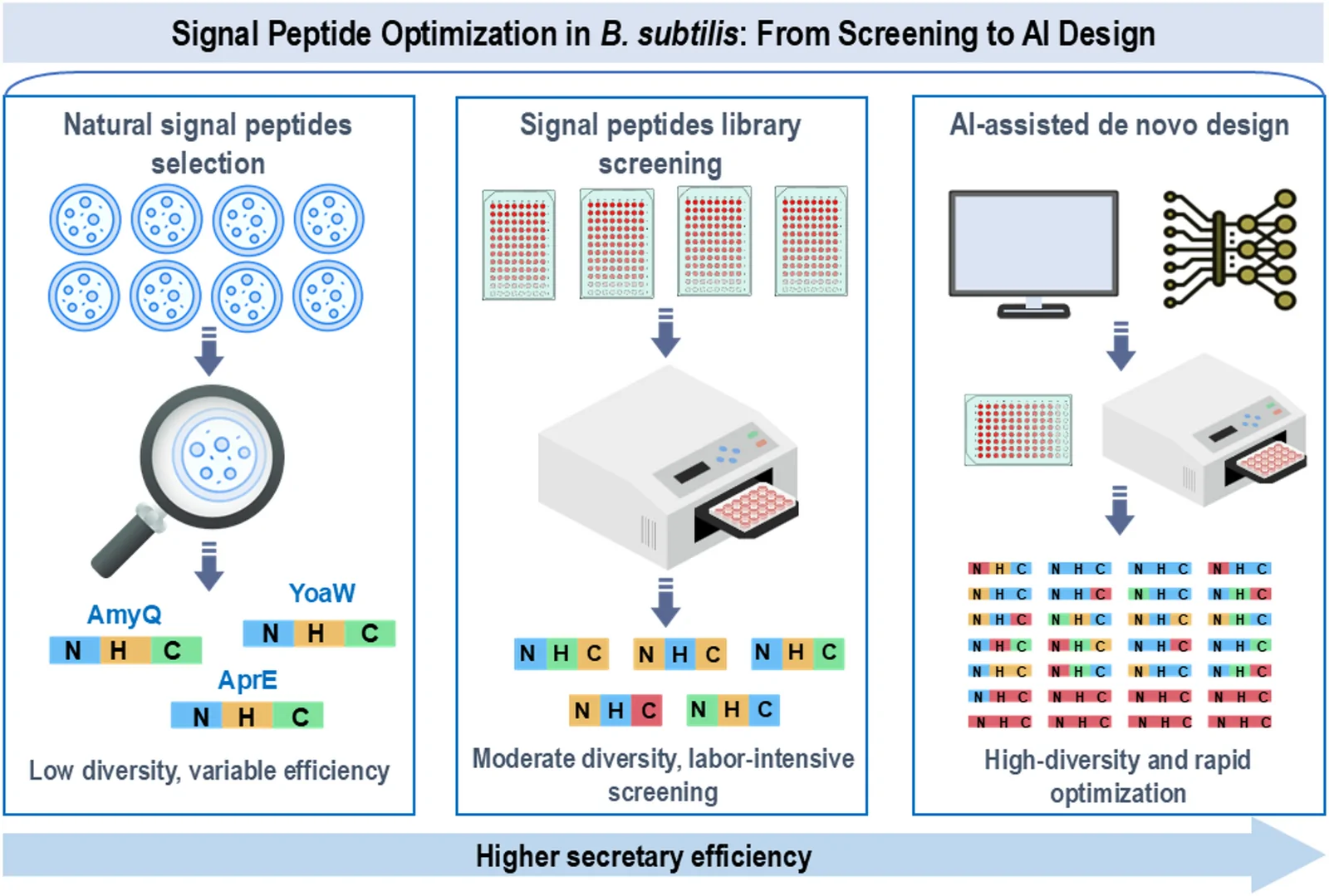
Schematic overview of SP optimization strategies in *B. subtilis*, illustrating the progression from natural SP selection to SP library screening and finally to AI-assisted de novo design, ultimately leading to enhanced protein secretion efficiency

SP engineering is increasingly moving beyond conventional library screening toward structure-guided, data-driven and generative design strategies that enable systematic optimization of N-, H- and C-region properties. Notably, Dai et al. introduced the SPgo framework, a hybrid computational approach combining rule-based design and deep generative modeling, which generated highly efficient Sec-type SPs that outperformed natural sequences and achieved secretion yields of up to 154 mg/L [80]. Complementarily, Kang et al. developed a transformer-based model trained on Gram-positive datasets and generated functional Sec/Tat SPs, with 15 of 16 experimentally tested candidates showing successful secretion, demonstrating the potential of data-driven SP design [86]. In parallel, Sun et al. developed SecEff-Pred, an ESM-2-based multitask learning framework for predicting SP secretion efficiency in *B. subtilis*, achieving up to ⁓86% prediction accuracy across multiple target proteins and highlighting the potential of protein language models for rational SP optimization [68]. Grasso et al. established a data-driven framework for the rational de novo design of SPs by screening ⁓12,000 engineered variants. By identifying the physicochemical determinants of secretion efficiency, the authors developed a predictive model that enables precise in silico optimization [77]. Li et al. developed a transfer-learning-based framework to design SPs in *B. amyloliquefaciens*, with 80% of ML-designed SPs enabling secretion and 60% achieving high-level secretion [87]. Collectively, these studies support the transition of SP engineering from empirical screening toward more predictable and generative design (Fig. 3).

**Table 2:** Comparative overview of computational tools and generative frameworks for signal peptide identification, cleavage site prediction and secretion efficiency optimization

Category	Tool / Framework	Algorithm / Approach	Training Data / Host Organism	Key Validated Performance / Capabilities
SP Prediction / Cleavage Mapping	SignalP 6.0 [81]	Transformer Language Model + Conditional Random Field (CRF)	All domains of life (Archaea, Bacteria, Eukarya)	Classification of five SP types (Sec/SPI, Sec/SPII, Tat/SPI, Tat/SPII, Sec/SPIII) and cleavage-site prediction
SP Prediction / Cleavage Mapping	DeepSig [83]	Deep Convolutional Neural Network (CNN)	Multi-organism benchmark datasets	Identification of Sec/SPI signal peptides and discrimination from N-terminal transmembrane segments in eukaryotes and prokaryotes
SP Prediction / Cleavage Mapping	Phobius [84]	Combined Hidden Markov Model (HMM)	Cross-species protein datasets	Combined prediction of signal peptides and transmembrane helices to avoid false positives
SP Prediction / Cleavage Mapping	PrediSi [85]	Statistical / machine learning sequence analysis	Annotated SP datasets	SP identification and cleavage-site mapping
SP Efficiency / De Novo Design	SPgo [80]	Hybrid rule-based / Deep Generative Modeling	Computationally designed / validated SPs	High-yield de novo Sec-type SP generation and optimization for heterologous expression
SP Efficiency / De Novo Design	Grasso et al. [77]	Physicochemical feature-based Machine Learning (156 features)	⁓12,000 engineered SP variants in *B. subtilis*	Quantifies feature importance (N-H-C region dynamics); predicts secretion efficiency of engineered variants
SP Efficiency / De Novo Design	SecEff-Pred [68]	ESM-2 Protein Language Model + Multitask Learning	*B. subtilis* secretion datasets	Predicts SP efficiency with high accuracy (⁓72–86% across α-amylase, alkaline xylanase, cutinase and phospholipase D)
SP Efficiency / De Novo Design	Kang et al. [86]	Transformer-based Generative Architecture	158,768 SP-protein pairs (Gram-positive bacteria)	De novo generation of target-tailored Sec and Tat signal peptides with high secretion yields
SP Efficiency / De Novo Design	Li et al. [87]	Transfer Learning-based ML Framework	*B. amyloliquefaciens* SP efficiency datasets	High-level SP design and secretion enablement for recombinant enzymes

Despite these advances, current AI-based prediction models still face several limitations. Most algorithms rely on annotated datasets that do not fully capture the diversity of SPs across bacterial species and are biased toward well-studied organisms, potentially limiting their generalization to industrially relevant *Bacillus* strains [81]. In addition, prediction performance can vary substantially among computational tools because of differences in model architecture, training datasets, and evaluation protocols [88]. Most importantly, many AI-based predictors have been evaluated primarily on curated or laboratory-scale datasets, whereas validation under industrial fermentation conditions remains limited [68]. Therefore, computational predictions should be regarded as complementary to, rather than replacements for, experimental validation.

### Promoter engineering for enhanced secretion

Promoter engineering is a pivotal strategy to optimize recombinant protein secretion in *B. subtilis*, as transcriptional strength directly dictates mRNA abundance and consequently, translation rate, secretion load and metabolic burden [89, 90]. Effective promoter design must balance high-level expression with the cell’s folding and translocation capacity to prevent stress responses and productivity loss.

Constitutive promoters drive continuous gene expression independently of external inducers. Widely employed examples include derivatives of P43 [91, 92] active during exponential and stationary phases, and P_*veg*_ [93, 94] active during vegetative growth. Engineered synthetic promoters with graded strengths further expand the toolkit. For example, the engineered P43’-riboE1 expression system achieved higher induced expression than the native promoters P43, P_srfA_, and P_aprE_, its performance depended on optimized regulatory architecture, including riboswitch design and spacer length [91]. Constitutive promoters are advantageous for industrial production due to their simplicity, cost-effectiveness and stable output. However, excessively strong constitutive expression can saturate the Sec pathway, leading to misfolded preproteins, secretion stress and leading to reduced growth rate [95].

Inducible promoters provide temporal control, allowing biomass accumulation before initiating production. Common systems include P_*xylA*_ (xylose-inducible) [94–96] and P_*spac*_ (IPTG-inducible) [96]. Inducible regulation is particularly valuable for proteins that are toxic, aggregation-prone or impose high secretion demand, as controlled induction mitigates early stress and enhances yield. Auto-inducible and stress-responsive promoters represent emerging strategies that exploit cellular physiology for dynamic expression control [97, 98]. For example, a recently engineered DegSU quorum sensing-responsive promoter (P_E742_) was developed by promoter engineering and regulatory circuit optimization, resulting in an 84.4% increase in regulatory strength. When implemented in an optimized *B. subtilis* chassis, this system increased acetoin production by 52.5% and extracellular carboxypeptidase activity by 65.9%, demonstrating the potential of auto-inducible promoters for high-level recombinant protein production under industrially relevant conditions [98]. These systems are activated by nutrient limitation, stationary phase signals or secretion cell envelope stress, enabling semi-autonomous regulation without external inducers and aligning expression with the cell’s folding and translocation capacity.

Balancing promoter strength with secretion capacity is critical. While strong promoters elevate mRNA levels, excessive transcription can overwhelm translation and translocation machinery, trigger stress responses such as the CssRS system, and reduce secretion efficiency [89, 99]. Intermediate promoter strength often achieves the highest yields by maintaining synthesis rates compatible with folding and export. Synthetic promoter libraries with defined strength variations facilitate systematic screening for optimal expression. These libraries are typically generated by modifying the − 35 and − 10 consensus regions, spacer length, UP elements and transcription factor binding sites. Rational pairing of promoter strength with SP identity and RBS efficiency enables fine-tuned expression, maximizing secretion without compromising cell viability [89, 90].

The integration of AI into metabolic and promoter engineering has enabled significant advances in de novo promoter design. Xia et al. developed PromoGen, a deep learning-based framework capable of generating species-specific prokaryotic promoters, even under limited data conditions. The model, built on pretrained multi-species nucleotide language models and fine-tuned for 27 individual species, successfully produced artificial promoters that closely mimic native sequence patterns in *E. coli* and *B. subtilis*. Comparative analyses demonstrated that PromoGen generated promoters outperforming previous neural network-based approaches, while experimental validation confirmed that all tested promoters were functional, with several exhibiting strong transcriptional activity [100].

Despite these promising results, current AI-driven promoter design platforms remain constrained by the availability and comprehensiveness of experimentally validated training datasets, which can limit model generalizability and increase the risk of overfitting to the training distribution [100]. Moreover, the robustness of AI-designed promoters under process-relevant fermentation conditions and their dynamic regulatory performance remain insufficiently characterized [98].

### Translational control via RBS optimization

Efficient recombinant protein secretion in *B. subtilis* is not determined solely by transcriptional strength or SP performance, but also critically depends on translational control. The RBS plays a central role in translation initiation and directly influences the rate at which nascent polypeptides are synthesized and delivered to the secretion machinery [101, 102]. In secretory expression systems, improper translational tuning can easily lead to secretion bottlenecks, protein misfolding and activation of stress responses. Therefore, RBS optimization has emerged as one of the key strategies for balancing protein synthesis with the cellular capacity for secretion [103]. In bacteria, translation initiation is primarily governed by the interaction between the Shine-Dalgarno (SD) sequence in the mRNA and the complementary anti-Shine-Dalgarno sequence at the 3’ end of the 16 S rRNA. In *B. subtilis*, the consensus SD sequence is similar to that of *E. coli* (AGGAGG), although considerable variation is tolerated. The strength of base pairing between the SD sequence and the 16 S rRNA strongly affects ribosome recruitment and consequently, translation initiation efficiency [104]. Equally important is the spacing between the SD sequence and the start codon (usually AUG). Optimal spacing in *B. subtilis* is typically 5–11 nucleotides, allowing proper positioning of the ribosome for accurate initiation [13, 101]. Deviations from this optimal distance can significantly reduce translation efficiency, even when a strong SD sequence is present. In addition, local mRNA secondary structures around the RBS and start codon can either facilitate or hinder ribosome access, further modulating translation rates. In secretory expression constructs, the RBS is functionally linked to the SP coding sequence. Changes in the RBS or the 5’ region of the coding sequence can alter mRNA folding patterns, influencing both translation initiation and early elongation. As a result, RBS design must be considered in the context of the downstream SP and target protein sequence.

Importantly, the relationship between RBS strength and secretion efficiency is not linear [105]. Increasing RBS strength initially enhances translation and can increase the supply of preproteins to the Sec machinery; however, when translation becomes excessive, it may increase intracellular precursor accumulation and place additional burden on the folding and secretion machinery. Conversely, excessively weak translation may limit protein production and underutilize the available secretion capacity. Thus, an intermediate translation rate is expected to provide a better balance between protein synthesis and secretion [101, 106]. This trade-off can be conceptually represented as a bell-shaped relationship between translation initiation rate and extracellular protein yield, with maximal production occurring within an optimal range of translation strength [101].

Rao et al. demonstrated that RBS engineering can provide a broad 104-fold dynamic range of gene expression in *B. licheniformis*, illustrating the large effect of translation initiation on protein production [105]. Complementarily, Liu et al. introduced the bsBETTER system, leveraging base editing to diversify genomic RBS sequences across multiple genes and generate combinatorial variants that enhanced metabolite production by up to 6.2-fold, while revealing strong context dependency of RBS strength [107]. However, increased translation does not necessarily result in a proportional increase in extracellular protein secretion. Fang et al. reported an approximately 20-fold increase in keratinase secretion through optimization of the 5’-UTR, including RBS and spacer engineering, highlighting the potential of translational tuning to improve secretion [108]. Together, these findings support the existence of a protein- and host-dependent optimum rather than a simple “stronger RBS is better” relationship.

### Combinatorial optimization of promoter, RBS and SPs

**Fig. 4:**
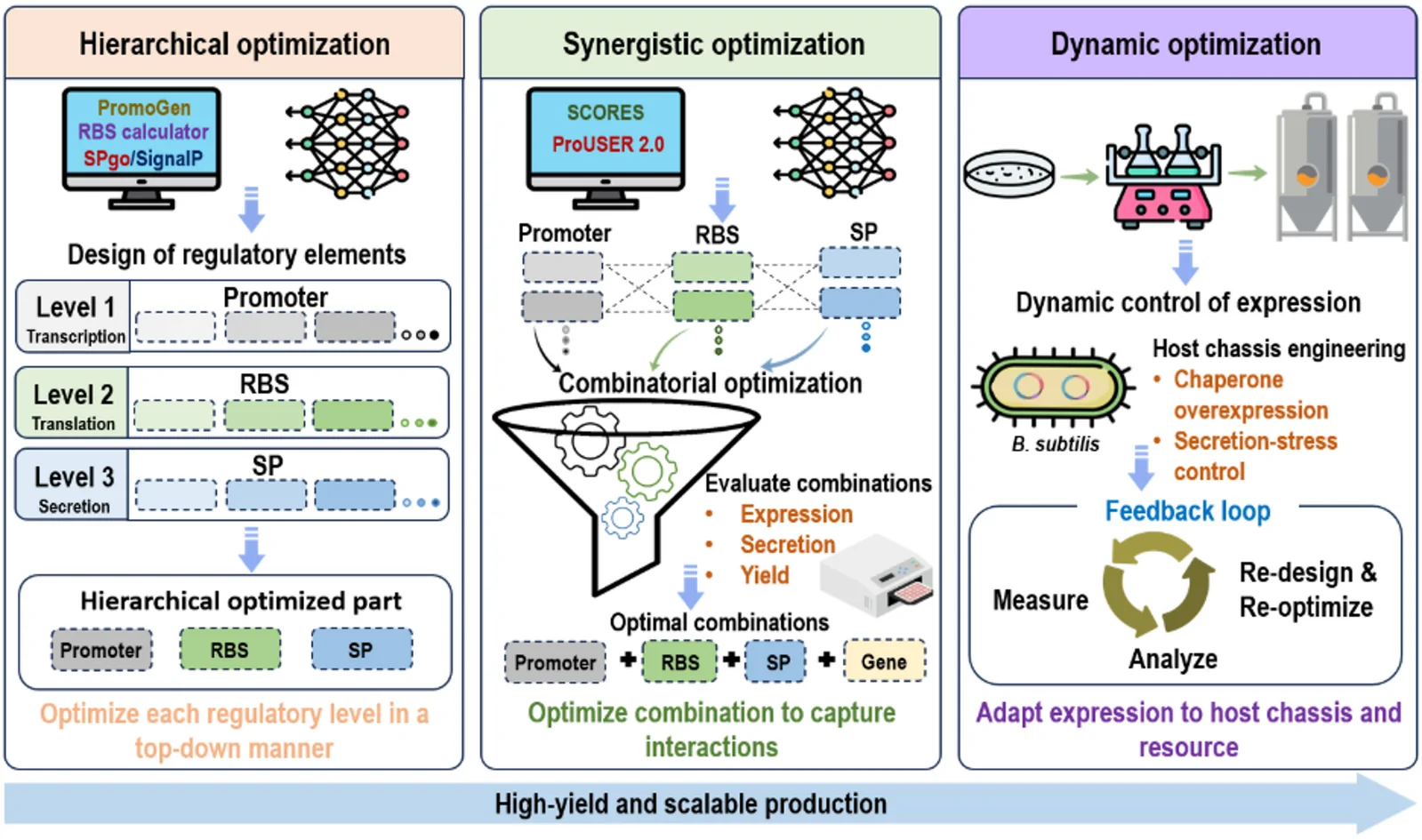
Unified framework integrating hierarchical, synergistic and dynamic optimization for microbial recombinant protein production. Hierarchical optimization coordinates regulatory elements across transcription, translation and secretion by systematically optimizing promoters, RBSs and SPs. Synergistic optimization considers the functional interactions among these elements and uses modular design, combinatorial screening and AI-assisted approaches to identify optimal promoter-RBS-SP combinations based on expression, secretion and production performance. Dynamic optimization adapts recombinant protein expression to host chassis and resource availability through feedback-based monitoring and iterative re-optimization. Together, these complementary strategies provide a systematic route toward high-yield and scalable recombinant protein production. This framework extends the established Design-Build-Test-Learn (DBTL) cycle by focusing specifically on the integrated optimization of regulatory elements, secretion processes and host responses

In the engineering of microbial recombinant protein production systems, increasing attention has been directed toward the combinatorial optimization of multiple regulatory elements to achieve more precise control over expression intensity, translation and protein secretion [109]. Promoters, RBSs, SPs and in some cases, transcriptional terminators collectively influence the synthesis, targeting and processing of recombinant proteins. Rather than optimizing these elements independently, their coordinated tuning can provide a broader means of balancing protein production with the folding and secretion capacity of the host cell [110, 111]. In *B. subtilis*, this integrated perspective is particularly relevant because transcriptional output, translation initiation and secretion efficiency are functionally coupled. To move beyond single-element optimization, we propose a unified framework integrating hierarchical, synergistic and dynamic optimization (Fig. 4). Hierarchical optimization coordinates regulatory elements across transcription, translation and secretion, including promoters, RBSs and signal peptides [110, 112]. Synergistic optimization recognizes that these elements interact and therefore should be optimized in combination [111], as excessive expression at one level may exceed downstream folding or secretion capacity. Dynamic optimization further adjusts expression according to cellular growth and resource availability, reducing metabolic burden and improving production stability [113].

Experimental studies provide evidence for these interaction effects. Li et al. demonstrated that combining a mini-cistron cassette, chaperone overexpression and SP screening increased soluble rhFGF21 secretion by ⁓9-fold [114], while Senger et al. and Wu et al. showed that secretion depended strongly on promoter-SP compatibility rather than on individual element strength [15, 72]. Similarly, Gan et al. demonstrated that coordinated optimization of promoter architecture and RBS strength can substantially enhance heterologous enzyme production, highlighting the importance of genetic-element compatibility [103]. Li et al. developed the SCORES strategy to systematically optimize RBS-signal peptide combinations, achieving a 7.5-fold increase in enzyme activity over the control combination [106]. Collectively, these studies illustrate hierarchical and synergistic interactions across expression and secretion layers rather than isolated effects of individual genetic elements.

The complex interplay among promoters, RBSs, SPs and host factors creates a large combinatorial design space, making exhaustive experimental screening increasingly challenging. Recent modular and high-throughput platforms provide practical approaches to address this complexity. ProUSER2.0 established a standardized modular platform with interchangeable genetic parts and signal peptide libraries for systematic engineering of protein expression and secretion in *B. subtilis* [94], while Matamouros et al. combined modular signal-peptide assembly with the AutoBioTech high-throughput platform to rapidly screen hundreds of constructs and identify protein-specific signal peptides in *C. glutamicum* [115]. These approaches illustrate how modular design and high-throughput screening can expand the exploration of combinatorial genetic space beyond conventional single-element optimization. Looking forward, integration of such experimental platforms with computational and AI-assisted design-build-test-learn strategies may further improve the efficiency of combinatorial optimization by prioritizing promising regulatory combinations for experimental testing. In this framework, experimental data from iterative screening could be used to refine computational predictions and progressively narrow the search space (Fig. 4). However, because secretion performance is strongly influenced by protein-specific and host-dependent factors, AI-based predictions should be regarded primarily as a tool for candidate prioritization rather than a substitute for experimental validation.

### Engineering the *B. subtilis* host chassis

Host chassis engineering provides a complementary strategy for improving heterologous protein production in *B. subtilis* by addressing bottlenecks in protein degradation, folding, secretion and cellular stress responses. One of the most established approaches is the deletion of extracellular proteases, including NprE, AprE, Epr, Bpr, Mpr, NprB, Vpr and WprA, which has led to protease-deficient production strains such as WB800N and KO7-S [116, 117]. Chassis optimization can also target the protein-folding machinery. In particular, the extracytoplasmic foldase PrsA has been shown to influence heterologous protein folding and secretion, while its performance depends on the signal peptide and promoter context. Combinatorial optimization of these components can therefore substantially affect recombinant protein yield.

A previous experimental study further illustrated the contribution of chassis engineering to recombinant protein production. The *B. subtilis* 168 htr9 ehp241 mutant, carrying an inserted hybrid prsA gene, increased the production of thermostable α-amylase from *B. licheniformis* 104.K by 1.14-fold relative to the htr9 host, demonstrating the potential of prsA-based chassis modification for improving recombinant enzyme production [20]. Consistent with this finding, Hamburger et al. demonstrated that the effects of PrsA depend on its combination with signal peptides and promoters, highlighting the importance of optimizing these components in a coordinated manner [118]. Similarly, Lu et al. used CRISPR-assisted regulation to activate prsA while repressing extracellular proteases, demonstrating that simultaneous manipulation of protein folding and degradation pathways can improve heterologous protein production [11].

Beyond protease and folding control, chassis engineering can target the secretion machinery itself. Systematic analysis of the Sec pathway has identified components of substrate targeting and translocation, including SRP/FtsY and SecYEG/SecA, as potential bottlenecks in heterologous protein secretion [119]. Such modifications provide a means of coordinating preprotein synthesis with the capacity of the secretion machinery. The CssRS two-component system represents another relevant control point because it senses secretion-associated cell-envelope stress and regulates downstream quality-control functions such as HtrA and HtrB [120]. Thus, rather than focusing on a single host modification, effective chassis engineering should coordinate protein degradation, folding, targeting, translocation and stress management to improve secretion performance.

### Bottlenecks and secretion stress

Despite extensive optimization of promoters, RBSs and SPs, recombinant protein secretion in *B. subtilis* can be limited by downstream folding, translocation and quality-control processes. High-level production of heterologous proteins can lead to the accumulation of misfolded or incompletely processed proteins at the membrane-cell wall interface, thereby triggering secretion stress and reducing productive secretion [121]. The severity of this response depends on both the expression level and the properties of the secreted protein, including its folding characteristics and interaction with the secretion machinery.

A central component of the secretion-stress response is the CssRS two-component system, which regulates the expression of the quality-control proteases HtrA and HtrB [120]. These proteases contribute to the removal of misfolded secretory proteins and therefore protect cells from excessive secretion stress; however, their activity can also contribute to degradation of recombinant products during high-level protein production [122]. Accordingly, reducing extracellular proteolysis, increasing folding capacity and adjusting expression levels represent complementary strategies for alleviating secretion stress.

An emerging strategy is to couple secretion-stress sensing with dynamic control of recombinant protein expression. A *B. subtilis* secretion-stress biosensor based on the CssRS-regulated htrA locus has recently been developed and combined with split-GFP-based monitoring to identify secretion bottlenecks and screen signal peptides while simultaneously assessing cellular stress [13]. Such systems provide a foundation for moving beyond static promoter optimization toward feedback-controlled expression, in which recombinant protein production could be adjusted according to the physiological state of the host. Although autonomous closed-loop control of secretion remains to be established in *B. subtilis*, integrating secretion-stress biosensing with tunable expression systems represents a promising direction for dynamic optimization of recombinant protein production.

### Future perspectives

Future development of recombinant protein secretion in *B. subtilis* will require integrated optimization of genetic elements and host physiology rather than isolated modification of individual components. Future high-throughput platforms could combine combinatorial libraries with automated screening to evaluate regulatory-element combinations across diverse recombinant proteins.

An important future direction is the integration of high-throughput experimental screening with data-driven modeling. Machine-learning models could learn sequence-phenotype relationships from standardized secretion datasets and prioritize signal peptides or combinations of regulatory elements for experimental testing. Because secretion phenotypes remain highly context-dependent, experimental validation will remain essential.

Dynamic regulation represents another promising direction. Secretion-stress biosensors based on the CssRS system provide a foundation for monitoring the physiological burden associated with recombinant protein production. Coupling such biosensors with tunable expression systems could enable feedback-controlled adjustment of recombinant protein synthesis according to the secretion capacity of the host. In addition, cell-free prototyping could provide a rapid intermediate platform for evaluating secretion-related genetic designs before their implementation in living *B. subtilis* cells. Together, these approaches could facilitate a transition from static optimization toward more predictive, adaptive and experimentally guided strategies for recombinant protein secretion.

## Conclusions

Recombinant protein secretion in *B. subtilis* is governed by the coordinated interplay of SPs, promoters and other regulatory elements, translational control, secretion machinery and host physiology. Rather than optimizing individual components independently, effective secretion requires synergistic control of expression, targeting, folding and proteolysis. Recent advances in combinatorial screening, host chassis engineering and AI-assisted design of promoters and SPs are moving the field toward more predictive and systematic optimization. Ultimately, integrating these approaches with dynamic control of secretion stress will be key to achieving robust, high-yield recombinant protein production in *B. subtilis*.

## Data Availability

No datasets were generated or analysed during the current study.

## Abbreviations

SP: Signal peptide
AI: Artificial intelligence
GRAS: Generally recognized as safe
PIPs: Posttranslationally interacting proteins
SRP: Signal recognition particle
PMF: Proton motive force
SPase: Signal peptidase
RBS: Ribosome binding sites
SD: Shine-dalgarno sequence
DBTL: Design-build-test-learn
